# Association between problematic smartphone use and ADHD symptoms among nursing students at a Saudi public university

**DOI:** 10.3389/fpsyt.2026.1776142

**Published:** 2026-06-03

**Authors:** Loujain Sharif, Jana Baeshen, Khadijah Abuhabaya, Dana Alsadik, Hala Alwagdani, Hala Elsayes, Amirah O. A. Alatawi, Ayman Mohamed El-Ashry

**Affiliations:** 1 Psychiatric and Mental Health Nursing Department, Faculty of Nursing, King Abdulaziz University, Jeddah, Saudi Arabia; 2 OR Department, Alsalama Hospital, Jeddah, Saudi Arabia; 3 Adult Emergency Department, King Abdulaziz Medical City, National Guard Health Affairs, Jeddah, Saudi Arabia; 4 OB/GYN Department, Johns Hopkins Aramco Healthcare, Khobar, Saudi Arabia; 5 Cardiac Intensive Care Unit (ICU) in King Faisal Cardiac Center, National Guard Hospital, King Abdulaziz Medical City, Jeddah, Saudi Arabia; 6 Psychiatric and Mental Health Nursing Department, Faculty of Nursing, Tanta University, Tanta, Al Gharbiyah Governorate, Egypt; 7 Therapeutic Program Department, Tabuk Health Cluster,Tabuk, Saudi Arabia; 8 Psychiatric and Mental Health Nursing, Faculty of Nursing, Alexandria University, Alexandria, Egypt

**Keywords:** attention-deficit/hyperactivity disorder, behavior, addictive, Saudi Arabia, smartphone, students, nursing

## Abstract

**Introduction:**

Problematic smartphone use is increasingly recognized as a behavioral pattern associated with adverse mental health outcomes, including attention-deficit/hyperactivity disorder (ADHD) symptoms. Understanding this relationship is particularly relevant among nursing students, who face substantial academic and clinical demands that may influence attention regulation and technology use patterns. This study assessed the proportion of students with higher-likelihood problematic smartphone use, the proportion screening positive on the Adult ADHD Self-Report Scale (ASRS-v1.1) Part A, and the association between these constructs among nursing students at a Saudi public university.

**Methods:**

A descriptive correlational cross-sectional design was used. A convenience sample of 221 nursing students completed an online self-administered questionnaire distributed through social media platforms. Measures included a sociodemographic questionnaire, the Arabic version of the Problematic Use of Mobile Phones (PUMP) scale, and the ASRS-v1.1 Symptom Checklist.

**Results:**

Greater problematic smartphone use was associated with greater ADHD symptom burden (r = 0.70, p < 0.001). In multivariable logistic regression, higher-likelihood problematic smartphone use was significantly associated with greater odds of screening positive on the ASRS-v1.1 Part A (OR = 3.26, 95% CI: 1.80–5.90; p < 0.001), whereas age, gender, academic level, and campus residence were not statistically significant or were imprecisely estimated.

**Discussion:**

Because the ASRS-v1.1 is a screening instrument and the PUMP categorization is operational rather than diagnostic, these findings should be interpreted as evidence of cross-sectional association rather than diagnostic prevalence. Longitudinal research is warranted to clarify directionality, mechanisms, and the role of key confounders in nursing education.

## Introduction

The education of nurses is not only stiff but also challenging, and it entails a prolonged focus, effective organizational abilities, and emotional stability during the academic and clinical training (1, 2). The nursing students must cope with numerous academic assignments, practice demands, deadlines, and care that may be emotionally stressful. Symptoms of attention-deficit/hyperactivity disorder (ADHD) are particularly pertinent to higher education as academic performance and student health can be adversely impacted by inefficiency in the regulation of attention, task switching, impulsiveness, and time management (3, 4). Even though the impairment is typically the central theme of ADHD, a growing body of literature also proposes the existence of such ADHD traits as creativity, high energy in activities of interest, and divergent problem-solving skills (5, 6). However, the high structure and demandingness of university education can render these issues of attention more conspicuous and more functional.

Meanwhile, smartphones have been firmly ingrained into the life of university students in terms of learning. Smartphones are traditionally employed in the context of nursing education (communication, access to lectures and course materials, organization of academic activities, access to educational resources, and apps) (7–9). Although these functions are convenient and flexible, the available nature of smartphones always can also be a source of distraction, compulsive checking, and excessive use patterns. The reasons include adverse psychological and behavioral effects of problematic smartphone use, the following of which include attentional difficulties, impulsivity, sleep disturbance, anxiety, and depression (10). An increased amount of literature has also suggested a positive correlation between ADHD symptomatology and problematic smartphone phone use in students and young adults (11, 12). These results imply that people who complain about more symptoms related to ADHD could also be more prone to exhibit problematic smartphone use patterns, but the direction of this association is not evident.

This association is especially relevant to nursing students. The ability to balance classroom and clinical demands, sustain concentration, maintain self-regulation, plan effectively, and manage competing tasks makes nursing education highly dependent on attention-related abilities. In that regard, co-occurring ADHD symptoms and problematic smartphone use may have meaningful implications for students’ academic functioning, mental health, and need for support services. Students with attentional challenges and problematic digital behaviors may struggle to complete coursework, remain focused during training, and manage competing academic and clinical demands. This is important because nursing students may already experience multiple sources of academic and clinical stress, and inclusive nursing education literature emphasizes the need for structured, non-stigmatizing support for students with attention-related or disability-related needs (13–16). Although studies on this association have increased in the general student population, there remains limited research on nursing students, who represent a group with unique academic and professional demands.

Specific research should also be conducted in the Saudi Arabian context. Cultural, educational, and technology-use patterns may influence smartphone-related behaviors, as well as the experience, interpretation, and reporting of attention-related symptoms. Previous studies have linked mobile-phone use, smartphone addiction, internet addiction, psychological well-being, depression, anxiety, stress, interpersonal problems, sleep quality, coping skills, mindfulness, and ADHD-related symptoms across adolescents, university students, and other young populations (17–22). At the same time, strength-based discussions of ADHD emphasize that attention-related traits may include hyperfocus, creativity, and resilience, although these strengths may coexist with functional difficulties in highly structured academic settings (23). Nevertheless, there is limited local evidence on how problematic smartphone use is related to ADHD symptom screening among university students, especially nursing students.

Based on this, this paper aimed to estimate the proportion of students with higher-likelihood problematic smartphone use, the proportion screening positive on the ASRS-v1.1 Part A, and the association between these constructs among nursing students at a Saudi public university. This evidence may help inform students' support efforts, mental health awareness, digital well-being initiatives, and future longitudinal research in nursing education.

### Objectives

1. To estimate the proportion of students with higher PUMP-classified problematic smartphone use.
2. To estimate the proportion of students screening positive on the ASRS-v1.1 Part A screener.
3. To examine the association between problematic smartphone, use and ADHD symptom severity among nursing students.

## Materials and methods

### Research design and setting

This study used a descriptive correlational, cross-sectional design using a self-administered questionnaire to collect data from nursing students at a Saudi public university. This design was selected to examine the association between problematic smartphone use and ADHD symptoms among nursing students. Ethical approval for this study was obtained from the Nursing Research and Ethics Committee (NREC) at the Faculty of Nursing of a Public University (NREC: Ref NO. 1f.22). Data collection was anonymous, and participation was voluntary, as no names or identifying information were collected. Detailed information about the study was provided on the first page of the online survey. Electronic informed consent was obtained from participants before they proceeded to complete the questionnaire. The study was conducted at the Faculty of Nursing of a public university established in 1977. The nursing program comprises a preparatory year, three academic years of coursework, and an internship year. Each academic level enrolls approximately 100–150 students. This setting was selected due to accessibility to the target population and a relatively large student body.

### Sample and sampling procedure

The study population included undergraduate nursing students enrolled at the Faculty of Nursing of a Saudi public university. Data were collected through an online survey distributed to students in the second, third, and fourth academic levels using a convenience sampling approach. *A priori* sample size calculation was conducted using G*Power for the planned primary analysis of the association between Problematic Use of Mobile Phone (PUMP) scores and Adult ADHD Self-Report Scale (ASRS-v1.1) scores using Pearson’s correlation (two-tailed). With α = 0.05, power = 0.80, and an anticipated moderate effect size based on prior literature examining the relationship between problematic smartphone use and ADHD symptoms in student populations (e.g., 24, 25), the minimum required sample was estimated at 220 participants. Among the 513 eligible students across these academic levels, 221 students participated and were included in the analysis.

### Measurement tools

Data were collected using a three-section questionnaire:

Section one: sociodemographic questionnaire.

This tool gathered demographic and background information, including participants’ age, gender, academic level, and living arrangement (on-campus or off-campus).

Section two: problematic use of mobile phone (PUMP) scale.

Problematic smartphone use was measured using the Arabic version of the Problematic Use of Mobile Phone (PUMP) Scale (26). The 20-item PUMP scale is aligned with DSM-5 substance-use criteria and has demonstrated good internal consistency and convergent validity in prior work (26, 27). Items are rated on a five-point Likert scale; higher total scores indicate more severe problematic smartphone use. Problematic smartphone use was measured using the Arabic version of the Problematic Use of Mobile Phone (PUMP) Scale (26). The 20-item PUMP scale is aligned with DSM-5 substance-use criteria and has demonstrated good internal consistency and convergent validity in Arabic-speaking and Saudi populations. Items are rated on a five-point Likert scale, yielding a total score from 20 to 100, with higher scores indicating greater problematic smartphone use. In the present study, the total PUMP score was analyzed as a continuous variable for descriptive statistics and correlation analysis. For descriptive categorical analyses only, participants were additionally classified into lower-likelihood and higher-likelihood problematic smartphone use groups using a score threshold of >61, as an operational cutoff used in prior Arabic-language work; this categorization should not be interpreted as a diagnostic threshold for smartphone addiction in this specific population.

Section three: adult ADHD self-report scale (ASRS-v1.1 symptom checklist).

ADHD symptoms were assessed using the Adult ADHD Self-Report Scale (ASRS-v1.1) Symptom Checklist (28), an 18-item self-report instrument developed in collaboration with the World Health Organization to assess adult ADHD symptoms based on DSM-IV criteria. The instrument consists of Part A (6 items), which serves as the screening component, and Part B (12 items), which provides additional information on symptom frequency. Each item was rated on a five-point scale from 0 (never) to 4 (very often), and responses across all 18 items were summed to generate a total symptom score ranging from 0 to 72, with higher scores indicating greater ADHD symptom burden. The ASRS total score was analyzed as a continuous variable for descriptive, correlational, and regression analyses. For descriptive screening purposes, participants were classified using the standard ASRS-v1.1 Part A rule: items 1–4 were scored as positive when responses were “sometimes,” “often,” or “very often,” whereas items 5–6 were scored as positive when responses were “often” or “very often.” Participants with 4 or more positive responses in Part A were classified as screening positive. This screening classification does not represent a clinical diagnosis.

### Validity and reliability of the instruments

Adult ADHD Self-Report Scale (ASRS-v1.1 Symptom Checklist) was translated and back-translated to maintain language and conceptual similarity of Arabic. The questionnaire was translated and a jury of three specialists in the field of psychiatric and mental health nursing was asked to review the questionnaire to test the clarity of the item, relevance, and suitability to the study purposes. Content validity was measured through item level content validity index (I-CVI) based on which the values obtained were between 0.80 and 0.99, which is acceptable and excellent content validity. According to the feedback regarding the work of the experts, some slight wording adjustments were done to make the work clearer and more understandable. Before final data collection, a pilot test was then carried out to determine feasibility, clarity and ease of administration.

The two psychometric scales used in the survey: Problematic Use of Mobile Phone (PUMP) scale containing 20 items and Adult ADHD Self-Report Scale (ASRS) containing 18 items were subjected to the reliability analysis to give a number of 38 scale items. Internal analysis of the sociodemographic questions was not covered in the analysis. The instruments were found to have high internal consistency with Cronbach alpha of 0.89 as was the case in the PUMP scale and 0.88 in the ASRS. The total Cronbach alpha on the 38 psychometric scale items was 0.934.

### Data collection procedure

Data were collected electronically through an online questionnaire distributed to eligible students. The participants were approached using online social media platforms including WhatsApp and X. The survey was comprised of a total of 42 items consisting of the demographics, PUMP, and ASRS. The questionnaire for data collection remained available online from March 2023 to April 2023. Participants were required to provide electronic informed consent before accessing and completing the questionnaire.

### Ethical considerations

Ethical approval for this study was obtained from the Nursing Research and Ethics Committee (NREC) at the Faculty of Nursing of a Public University. Data collection was anonymous, and study enrolment was voluntary, as the participants were not asked to include their names or any identifying information. Detailed information about the study was included on the first page in the online survey. Therefore, before the participants started the survey, they had the chance to read the information provided and decide whether they were interested in being enrolled in the study. Electronic informed consent was obtained from all participants prior to survey completion.

### Data analysis

Statistical Package of the Social Sciences (SPSS) version 28 was used to analyze the data. The frequencies, percentages, means, and standard deviations summarized the sociodemographic characteristics and the study variables of the participants. Means and standard deviations were used to summarize continuous PUMP and total ASRS scores and provide the basis of descriptive and correlational analysis. Since the two variables were measured using summed multi-item scales and current as approximately continuous, Pearson correlation coefficient was employed to test the relationship between problematic smartphone use and total symptom score on ADHD. To assess the robustness of the association, a sensitivity analysis using Spearman’s rank correlation was also conducted.

Frequencies and percentages were used to summarize categorical variables. To perform descriptive categorical analyses, the participants were divided into PUMP categories (>61 vs. 061) and ASRS-v1.1 Part A screen-positive or screen-negative based on the standard ASRS-v1.1 Part A rule. The tests of bivariate associations were tested with the help of chi-square tests in the event of meeting the assumption. Because the oldest age category has only a single participant, age was pooled into 1822 years and 2330 years to conduct categorical analyses to be sure that the expected cell count is sufficient. Fisher explicit test was applied where the anticipated counts of cells were too small. Multivariable logistic regression was undertaken to investigate variables that may be linked to ASRS-v1.1 screening positivity. Problematic smartphone use and sociodemographic characteristics were the independent variables, whereas ASRS-v1.1 screening positivity was the dependent variable in the regression model. The odds ratios (ORs) were reported to be based on 95 percent confidence intervals (CIs) and the level of statistical significance was set at p < 0.05.

Since both PUMP and ASRS were multi-item scale scores summed and approximated to be continuous variables, Pearson correlation coefficient was employed in testing the relationship between them.

## Results

Out of 221 total nursing student respondents most of them (91%) were aged 18–22 showing that the sample was predominately young, and the majority (81%) were female. Because the oldest age category contained only one participant, age categories collapsed for categorical analyses when needed to satisfy chi-square assumptions. The sample distribution by academic level was almost equal between years two, three and four. Most students lived off campus (94%). This profile suggests a relatively even distribution on age and residence, with gender imbalance typical of nursing programs (see **Table 1**).

**Table 1:** Distribution of nursing students’ characteristics (n=221).

Variables	Categories	N	%
Age	18 - 22	202	91
	23 - 30	19	9
Gender	Male	42	19
	Female	179	81
Academic level	Second	80	36
	Third	67	30
	Fourth	74	33
Do you live on campus?	Yes	13	6
	No	208	94

Mean PUMP score was 64.23 (SD 14.43), and mean ASRS score was 40.10 (SD 12.60), indicating substantial variability on both measures (**Table 2**).

**Table 2:** Distributions of mean and standard deviation of problematic use of mobile phone (PUMP) scale and Adult ADHD self-report scale (ASRS) (n=221).

Study variables	Mean	SD
Total mean score of Problematic use of mobile phone	64.23	14.43
Total mean score of Adult ADHD self-report scale (ASRS)	40.1	12.6

**Figure 1:**
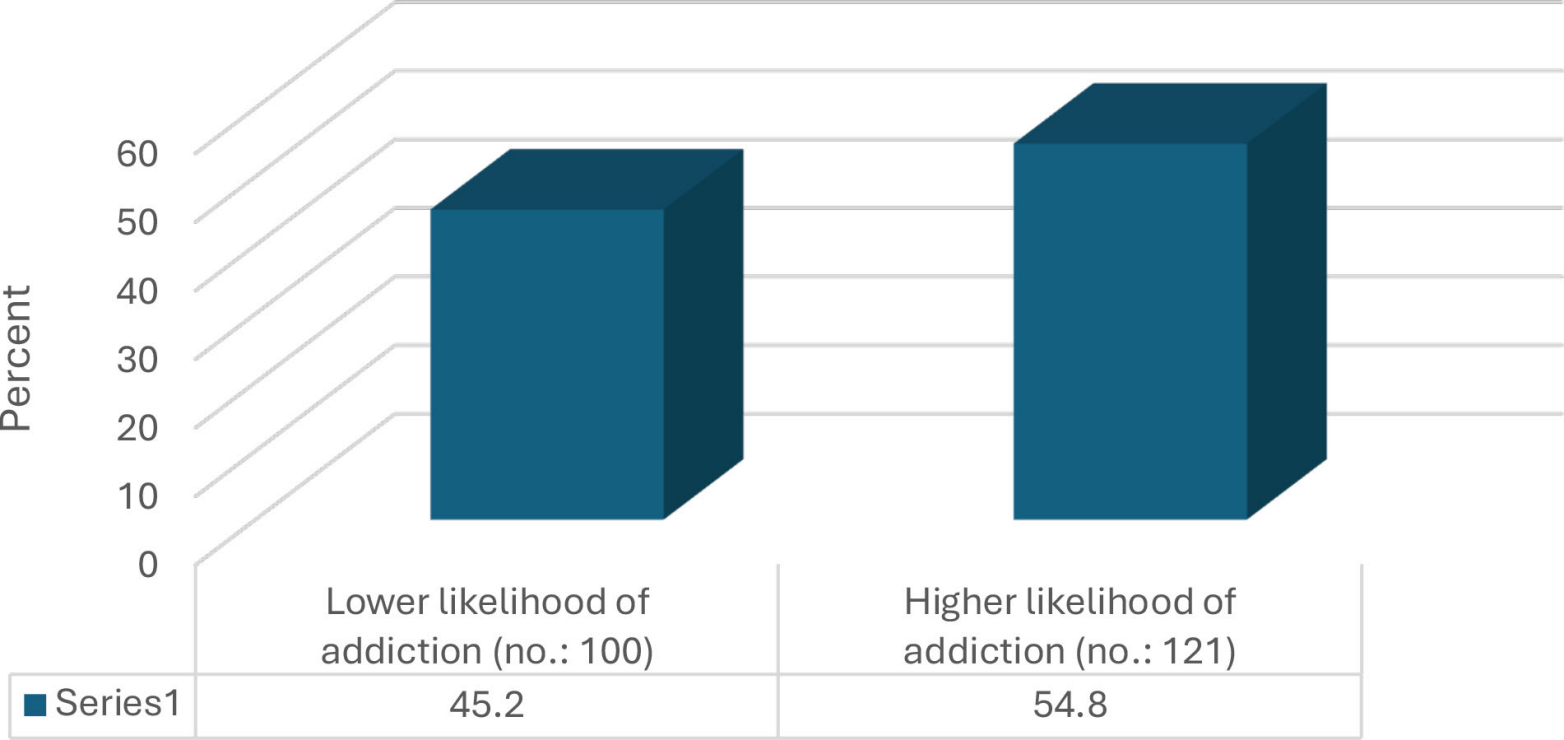
Shows the proportion of participants classified in the higher-likelihood problematic smartphone use group.

**Figure 1** shows the proportion of participants classified in the higher-likelihood problematic smartphone use group. Over half of the cohort were classified in the higher-likelihood problematic smartphone use group, indicating that problematic use is common in this setting.

**Figure 2:**
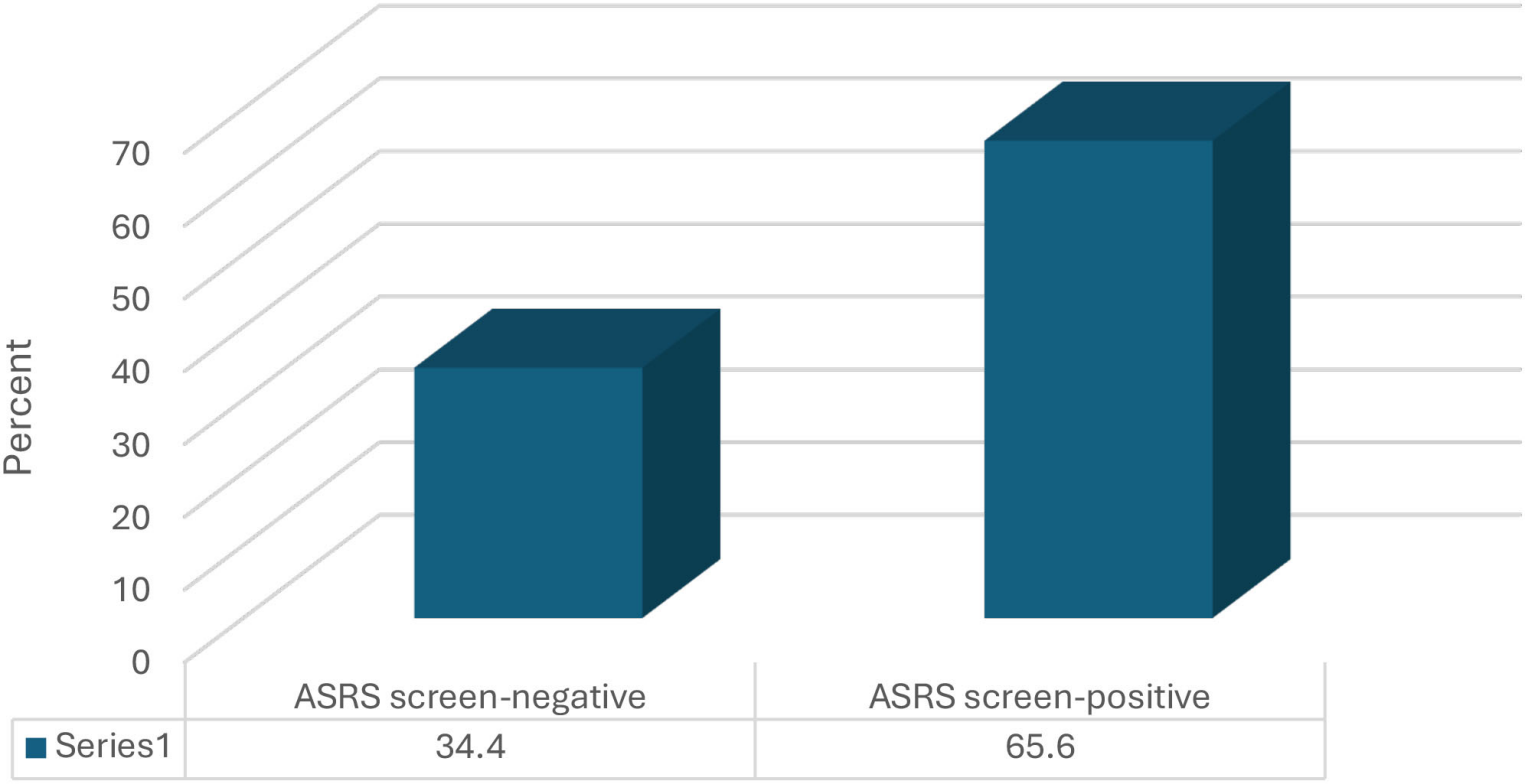
Percentage distribution of ASRS-v1.1 part A screening status.

**Figure 2** shows the percentage distribution of participants according to ASRS-v1.1 Part A screening status. A substantial proportion of students screened positive on the ASRS-v1.1 screener. Because the ASRS-v1.1 is a screening instrument, these findings reflect screening positivity rather than clinically confirmed ADHD. **Figure 2** shows the proportion of participants who screened positively on the ASRS-v1.1 Part A using the standard rule. Overall, 145 of 221 students (65.6%) screened positive and 76 (34.4%) screened negative. These values reflect screening status only and should not be interpreted as diagnostic prevalence of ADHD.

The relationship between problematic smartphone use and participants’ characteristics is shown in **Table 3**. Problematic smartphone use differed significantly by gender (χ² = 5.82, p = 0.016), with a higher proportion of males classified in the higher-likelihood problematic smartphone use group than females. No significant differences were observed by age, academic level, or campus residence (all p > 0.05). ASRS-v1.1 screening status was significantly associated with problematic smartphone use (χ² = 17.28, p < 0.001), indicating a strong cross-sectional relationship between problematic smartphone use and ADHD screening positivity.

**Table 3:** Relationship between problematic smartphone use and nursing students’ characteristics (n=221).

Variables	Categories	Lower likelihood of problematic smartphone use n (%)	Higher likelihood of problematic smartphone use n (%)	χ²	p-value
Age	18 - 22	93 (93.0)	109 (90.1)	0.28	0.597
	23 - 30	7 (7.0)	12 (9.9)		
Gender	Male	12 (12.0)	30 (24.8)	5.82	0.016*
	Female	88 (88.0)	91 (75.2)		
Academic level	Second	38 (38.0)	42 (34.7)	0.28	0.869
	Third	29 (29.0)	38 (31.4)		
	Fourth	33 (33.0)	41 (33.9)		
Do you live on campus?	Yes	6 (6.0)	7 (5.8)	0.005	0.946
	No	94 (94.0)	114 (94.2)		
ASRS-v1.1 screening status	Screen negative	49 (49.0)	27 (22.3)	17.28	<0.001*
	Screen positive	51 (51.0)	94 (77.7)		

*Statistical significance was set at p < 0.05.

Because the 27–30-year age category contained only one participant, age categories were collapsed into 18–22 and 23–30 years for categorical analyses. Fisher’s exact test was used when expected cell counts were too small for chi-square assumptions.

**Table 4** shows the relationship between ASRS-v1.1 part A screening status and participants’ characteristics. Because the oldest age category contained only one participant, age was collapsed into 18–22 years and 23–30 years for categorical analyses. No statistically significant associations were observed between ASRS screening positivity and age, gender, or academic level (all p > 0.05). Campus residence was associated with ASRS screening status when assessed using Fisher’s exact test (p = 0.038), reflecting the small number of students living on campus.

**Table 4:** Relationship between ASRS-v1.1 part A screening status and nursing students’ characteristics (n=221).

Variables	Categories	ASRS-v1.1 Part A		χ2	p-value
		Screen negative	Screen positive		
Age	18-22	70 (92.1)	132 (91.0)	0.00	0.986
	23-30	6 (7.9)	13 (9)		
Gender	Male	10 (13.2)	32 (22.1)	2.03	0.155
	Female	66 (86.8)	113 (77.9)		
Academic level	Second year	31 (40.8)	49 (33.8)	1.30	0.523
	Third year	20 (26.3)	47 (32.4)		
	Fourth year	25 (32.9)	49 (33.8)		
Do you live on campus?	Yes	1 (1.3)	12 (8.3)	Fisher’s exact test	0.038*
	No	75 (98.7)	133 (91.7)		

*Statistical significance was set at p < 0.05.

Because the 27–30-year age category contained only one participant, age categories were collapsed into 18–22 and 23–30 years for categorical analyses. Fisher’s exact test was used when expected cell counts were too small for chi-square assumptions.

**Table 5** presents both the primary Pearson correlation and the Spearman sensitivity analysis for the association between PUMP scores and total ASRS scores. Pearson’s correlation showed a strong positive association (r = 0.70, 95% CI: 0.62–0.76, p < 0.001). In sensitivity analysis, Spearman’s rank correlation showed a similar result (ρ = 0.64, 95% CI: 0.55–0.71, p < 0.001), supporting the robustness of the findings.

**Table 5:** Pearson correlation and Spearman sensitivity analysis for the association between PUMP scores and total ASRS scores among nursing students (n = 221).

Analysis	Coefficient	5% CI	p-value
Pearson correlation	r = 0.70	0.62–0.76	<0.001*
Spearman rank correlation (sensitivity analysis)	ρ = 0.64	0.55–0.71	<0.001*

PUMP, Problematic Use of Mobile Phone; ASRS, Adult ADHD Self-Report Scale. Pearson correlation was the primary analysis because both variables were analyzed as continuous summed scale scores. Spearman’s rank correlation was conducted as a sensitivity analysis to assess the robustness of the association. Statistical significance was set at *p < 0.05.

**Table 6** presents the multivariable logistic regression analysis of factors associated with ASRS-v1.1 Part A screening positivity. After adjustment for age, gender, academic level, and campus residence, higher-likelihood problematic smartphone use remained significantly associated with greater odds of screening positive on the ASRS-v1.1 (B = 1.18, OR = 3.26, 95% CI: 1.80-5.90, p < 0.001). Campus residence showed a borderline association, with students living on campus having higher odds of ASRS screening positivity than those living off campus (B = 2.09, OR = 8.10, 95% CI: 1.00-65.56, p = 0.050). No statistically significant associations were observed for age (B = -0.07, OR = 0.93, 95% CI: 0.31-2.83, p = 0.901), gender (B = 0.50, OR = 1.65, 95% CI: 0.73-3.70, p = 0.228), or academic level (B = 0.06, OR = 1.06, 95% CI: 0.74-1.54, p = 0.736). Model diagnostics indicated acceptable fit and modest discrimination (-2 Log Likelihood = 259.43, Cox & Snell R² = 0.107, Nagelkerke R² = 0.148, AUC = 0.680, classification accuracy = 68.3%).

**Table 6:** Multivariable logistic regression analysis of factors associated with ASRS-v1.1 Part A screening positivity (n = 221).

Variable	Coding/reference category	B	Wald	p-value	Odds ratio (95% CI)
Age	23–30 years vs 18–22 years	-0.07	0.02	0.901	0.93 (0.31–2.83)
Gender	Male vs Female	0.50	1.45	0.228	1.65 (0.73–3.70)
Academic level	Per one-level increase (Second → Third → Fourth year)	0.06	0.11	0.736	1.06 (0.74–1.54)
Do you live on campus?	Yes vs No	2.09	3.85	0.050	8.10 (1.00–65.56)
Problematic smartphone use	Higher-likelihood (PUMP > 61) vs Lower-likelihood (PUMP ≤ 61)	1.18	15.15	<0.001	3.26 (1.80–5.90)

-2 Log Likelihood = 259.43; Cox & Snell R² = 0.107; Nagelkerke R² = 0.148; model likelihood-ratio p = 0.00014; AUC/ROC = 0.680; classification accuracy = 68.3%.

## Discussion

This study has looked at the relationship between problematic smartphone use and symptom burden of ADHD in a sample of nursing students in a state university in Saudi Arabia when assessed by continuous ASRS scores and traditional ASRS-v1.1 Part A screening rule. The correlation analysis revealed that the relationship between PUMP and ASRS total scores was significant (r = 0.63, p < 0.001). At the point where the normal ASRS screening rule was used, 65.6% of nursing students screened positive on the ASRS-v1.1 Part A. This percentage must be viewed critically because ASRS screening in this sample is positive and not diagnostic prevalence of ADHD. Similarly, the PUMP grouping represents an operational category of problematic smartphone use of higher likelihood in contrast with a clinical diagnosis. In bivariate analysis, a positive ASRS screening status was related to the problematic use of the smartphone, which was statistically significant even after the demographic variables that could influence the correlation were controlled. Based on this, the continuous-score correlation was stronger as compared to the adjusted binary-screening model.

Results have shown that problematic smartphone use was positively associated with the burden of ADHD symptoms and ASRS-v1.1 Part A screening positivity in this cohort. In the adjusted analysis, students in higher likelihood problematic smartphone use group were more likely to screen positive on the ASRS-v1.1 Part A (OR 3.26, 95% CI: 1.80- 5.90; p <.001). These findings cannot be understood to reflect clinically validated prevalence or diagnosis of ADHD; instead, the same implies cross-sectional co-occurrence of high problematic smartphone use with higher symptom burden or screening positivity of ADHD. This trend aligns with the previous sources of evidence of links between problematic smartphone use and ADHD symptoms or ASRS screening positivity (10, 12, 25). The differentiation between these two constructs in this sample was significant (r = 0.63), indicating that there was substantial co-occurrence between the two constructs.

Abnormalities in sustained attention, inhibition and reward sensitivity associated with ADHD may be associated with a tendency toward to repetitive checking and immediate digital feedback as a reward. On the other hand, excessive smartphone use may be associated with increased attentional fragmentation, decrease the ability to sustain attention to tasks, and disrupt cognitive control, which leads to a vicious cycle of attentional problems and problematic use of digital devices (12, 24, 25, 29). The current cross-sectional design does not make it possible to establish causal direction and mechanisms.

The gender difference in problematic smartphone use was significant with males being more likely to be in the high-likelihood group. This is not the first report on gender disparities in digital usage behavior and other related factors, especially in the context of gaming, online entertainment, and strongly reinforcing smartphone activities (12, 24). The most demographic characteristics did not significantly differ in terms of ASRS-v1.1 screening positivity, implying that the latter was widely distributed across subunits in this sample. Nevertheless, in the current study several probable confounders were not captured such as the quality of sleep, anxiety and depression, stress and academic workload, substance use, previous diagnosis and/or use of ADHD medication, and objective smartphone use time. These immeasurable variables can partially resolve the apparent association and must be included in future research and be taken into consideration on the interpretation of the current results.

In our study, problematic smartphone use was the strongest independent variable associated with positive ASRS screening status after controlling for demographic variables, consistent with prior research demonstrating a robust association between problematic smartphone use and positive ASRS screening status (24, 25). This finding is particularly relevant in nursing education, where high cognitive and organizational demands may interact with attentional vulnerabilities and problematic digital behaviors to affect student well-being and functioning (10). Importantly, this study assessed problematic smartphone use rather than academic/functional smartphone use; therefore, findings should not be interpreted as evidence against educational applications of mobile technology (7, 8). Future studies should distinguish academic from non-academic smartphone use and evaluate potential mediators (e.g., sleep quality, stress, psychological distress) and confounders (e.g., academic workload) (12, 25).

## Limitations

There are also several limitations to this study which ought to be considered when the results are being interpreted. To begin with, the cross-sectional design does not allow the causal inference between the problematic smartphone use and the ADHD-related symptoms, thus, the directionality and driving mechanisms of the studied associations cannot be identified. Second, convenience sampling and online recruitment using social media sites could have caused self-selection bias. Third, a small sample was used, and it was selected among the nursing faculty of one Saudi public university which restricts its generalizability to other groups of students and environments. Fourth, the data were all self-reported and thus liable to the recall and social desirability bias. Fifth, a few key possible confounders were not measured, such as sleep quality, anxiety and depression, stress and academic workload, substance use, previous ADHD diagnosis or medication, and objective smartphone use time, thus residual confounding remains likely. Moreover, symptoms of ADHD have been measured with the help of the ASRS-v1.1 screening instrument as opposed to a diagnostic one, and the PUMP cutoff has been employed as an operational categorization as opposed to a diagnostic cutoff; thus, the given results are a measure of screening positivity and problematic-use classification instead of a diagnostic one. The confidence intervals of some logistic regression estimates, especially on the residence of the campus, were wide and probably because of the sparse data in some categories, thus, the odds ratios should be interpreted critically.

Future research should address these limitations by employing longitudinal designs, multi-site sampling, and multi-method approaches, including objective measures of smartphone use and structured clinical assessments of ADHD where feasible.

## Conclusions

This research identified a significant percentage of students classified in the higher-likelihood problematic smartphone use group and a high proportion of students who screened positive to the ASRS-v1.1 among nursing students in a government university in Saudi Arabia. The problematic smartphone use was significantly and positively associated to the symptom burden of ADHD and was also the strongest independent predictor of a positive result in the ASRS screening when adjusted by the demographic factors. The results substantiate the necessity of awareness, screening-based student support intervention, and additional longitudinal research based on diagnostic evaluation, objective behavioral measures, and critical confounder evaluation.

## Data Availability

The original contributions presented in the study are included in the article/supplementary material. Further inquiries can be directed to the corresponding author.
